# Trends in chronic kidney disease-related mortality in older adults in the United States from 1999 to 2020: a cross-sectional study

**DOI:** 10.1097/MS9.0000000000003822

**Published:** 2025-09-12

**Authors:** Inshal Jawed, Ahsan Feroze, Maheen Zahid, Muhammad Ahmad, Shahzeb Humayun Kaleem, Sheema Saadia, Madiha Salman, Muhammad Khuzzaim Khan, Marium Mehmood, Safia Rashid, Ahmed Kamal Siddiqi, Salma S. Alrawa, Shafaq Jabeen, Danaish Kumar, Rahul Rai

**Affiliations:** a Department of Medicine, Dow University of Health Sciences, Karachi, Pakistan; b Department of Medicine, Jinnah Medical and Dental College, Karachi Pakistan; c Department of Medicine, Liaquat University of Medical and Health Sciences, Jamshoro, Pakistan; d Department of Medicine, Khyber Medical College, Peshawar, Pakistan; e Department of Medicine, Aga Khan University Hospital, Karachi, Pakistan; f Division of Cardiothoracic Imaging, Department of Radiology and Imaging Sciences, Emory University, Atlanta, GA, USA; g Faculty of Medicine, University of Khartoum, Khartoum, Sudan; h Department of Medicine, Karachi Medical and Dental College, Karachi, Pakistan

**Keywords:** chronic kidney disease, mortality, older adults, united states

## Abstract

**Background:**

Chronic kidney disease (CKD) is becoming increasingly common in the United States, particularly among older adults. However, little is known about the mortality patterns within this demographic.

**Objective:**

This study aims to explore the demographic and regional patterns of CKD-associated mortality in the United States from 1999 to 2020 among adults >65 years old.

**Methods:**

The Centers for Disease Control and Prevention’s WONDER (Wide-Ranging Online Data for Epidemiologic Research) database’s death certificates were analyzed for CKD-related mortality in persons aged 65 and older between 1999 and 2020. Age-adjusted mortality rates (AAMRs) per 10 000 and annual percentage change were calculated, stratified by geographic region, sex, year, and race/ethnicity.

**Results:**

Between 1999 and 2020, 1 572 075 CKD-related deaths occurred among adults ≥65 years. AAMR increased significantly from 132.0 in 1999 to 216.6 in 2020. Men consistently had higher AAMRs than women. Non-Hispanic (NH) Black has the highest AAMR (312.3), followed by NH American Indian (188.8), Hispanic (178), NH White (157), and NH Asian (143.9). Additionally, there was significant regional heterogeneity in AAMR (Midwest 184.6; South: 171.6; West: 165.5; Northeast: 157.4), with CKD-related AAMR being greater in non-metropolitan areas. States with CKD-related rates in the top 90th percentile AAMR were West Virginia, North Dakota, Indiana, Ohio, South Carolina, and North Carolina.

**Conclusions:**

CKD-related mortality in US adults ≥65 years has increased from 1999 to 2020. The highest AAMRs were noticed among NH Blacks, men, and patients residing in non-metropolitan and Midwestern areas of the United States. A targeted approach is required to significantly decrease the mortality in this population group.

## Introduction

Chronic kidney disease (CKD) has emerged as a major global health challenge, with a notable increase in both prevalence and mortality. It is estimated that CKD affects >10% of the world’s population which amounts to >800 million individuals^[1]^. Almost third of CKD patients live in China and India^[2]^. Between 1990 and 2017, the global all-age mortality rate from CKD rose by 41.5%, making it the 12th leading cause of death worldwide^[2]^. This upward trend is driven in part by the rising incidence of major risk factors such as diabetes, hypertension, and obesity^[3,4]^. For example, approximately 24% of CKD cases are associated with diabetes^[5]^, and CKD is present in about 20% of adults with hypertension^[6]^. The prevalence of CKD among US adults with hypertension is three times that of those without hypertension^[7]^. Similarly, one in every three with diabetes have CKD^[8]^ and 39.1% of CKD patients in the United States have diabetes or prediabetes^[9]^. During 2021–2023 the prevalence of these risk factors amongst the US adult population aged 60 and above was 27.3%, 38.9%, and 71.9% respectively, which partially explains the rising burden of CKD among the US adults^[10–12]^. These conditions not only increase the risk of developing CKD but also contribute to more rapid disease progression and higher mortality rates in this age group. Despite this, CKD awareness remains critically low, with only 6% of the general public and 10% of high-risk individuals globally being aware of their CKD status^[13]^.

The global population is aging, and as with other chronic diseases, augments the risk of developing CKD. In 2030, 37.8% of US individuals 65 and older are expected to have CKD^[14]^. In addition, it is projected that by 2030, the population will be 73 million people in the United States who are 65 years or older^[15]^. The number of older people with CKD is expected to rise significantly as a result of this combination, leading to corresponding increases in healthcare expenses and a decline in quality of life.

Regional and sociodemographic differences in CKD mortality and prevalence are suggested by recent data^[16,17]^. Racial minorities and individuals with low economic status are affected disproportionately and have faster disease progression^[18,19]^.

Identifying at-risk populations is vital for implementing a targeted approach to reducing disease mortality and morbidity. Therefore, this study aims to explore the demographic and regional patterns of CKD-associated mortality in the United States from 1999 to 2020 among adults >65 years old.

## Methodology

Statistics on chronic renal disease-related mortality in the US population over 65 years old were gathered using the Centers for Disease Control and Prevention (CDC) WONDER (Wide-Ranging Online Data for Epidemiologic Research) database. The CDC created CDC WONDER, an integrated public health information and communication system. It makes the CDC’s vast online data source for epidemiologic research accessible to the general public and public health professionals via a menu-driven interface. As we used this publicly available, deidentified data, ethical approval was not needed.

The Multiple Cause of Death data set contains population and mortality statistics for each county in the United States. The Division of Vital Statistics, the National Center for Health Statistics, the CDC, the US Department of Health and Human Services, and the Mortality Statistics Branch generate the data set that is used to create the Multiple Causes of Death data. The Vital Statistics Cooperative provides the data from the 57 vital statistics jurisdictions.

Death certificates for US citizens provide the basis of the data. Each death certificate includes 1 underlying cause of death, up to 20 additional reasons for death, and demographic data. Since CKD is strongly correlated with multiple underlying comorbidities, we extracted data from the subset of various causes of death to better accommodate the size effect and include all the patients having CKD as a comorbidity.

The number of deaths, crude mortality rates, age-adjusted mortality rates (AAMRs), annual percentage change (APC), and 95% confidence intervals (CIs) for mortality rates were calculated using the following factors: causes of death, place of residence (national, region, division, state, and county), age, race (American Indian or Alaskan Native, Asian/Pacific Islander, Black or African American, White), gender, and year. Missing data were handled according to CDC WONDER’s standard suppression rules, where counts less than 10 were suppressed to protect confidentiality. We excluded suppressed or missing categories from the final analysis. No imputation methods were applied, and only complete records with identified demographic variables and cause of death were included. The trends were then created using JoinPoint and analyzed to assess the pattern of mortality related to CKD, better understand its social dynamics, and aid in making future trajectories for utilizing health-related resources via public health policies.

**HIGHLIGHTS**

- Chronic kidney disease (CKD)-related mortality has increased in the United States from 1999 to 2020, and age-adjusted mortality rose from 132.0 to 216.6 per 100 000.
- CKD mortality is higher in men. Non-Hispanic (NH) Black or African Americans have the highest mortality, with an increase in NH Whites.
- Mortality in non-metropolitan areas surpassed metropolitan areas, likely due to limited access to specialist care and delayed intervention.
- Arizona had the lowest age-adjusted death rate, while West Virginia had the highest. Southern states showed a heavy CKD burden.
- There is a need for targeted strategies to reduce CKD-related mortality, especially among racial minorities, older adults, and rural populations.

The study was conducted in compliance with updated STROCSS Guideline^[20]^.

### Inclusion and exclusion criteria

The study included all the datasets from the database for individuals greater than 65 years of age with CKD-related mortality between 1999 and 2020. CKD-related deaths were identified using International Classification of Diseases and Related Health Problems – 10th Revision codes N18.0–N18.9 listed as any of the multiple causes of death on the death certificate. To ensure inclusiveness, we analyzed deaths where CKD was listed among the contributing causes, not just as the underlying cause, as CKD often coexists with other chronic conditions. There were no limitations regarding gender or place of residence. All pertinent cases during the allotted period were included to create a representative sample for the study. Nonetheless, the analysis did not include the cases with unidentified categories and suppressed data.

### Variables and measures

The main outcome measure of interest was to analyze major trends and patterns related to the CKD-related mortality rate through the socioeconomic, genetic, and geographic variables determined per 100 000 individuals. The analysis concentrated on years with the highest mortality rates to further understand and explore temporal variations. Potential confounding factors such as socioeconomic status, healthcare accessibility, and comorbidities were not directly available in CDC WONDER and thus could not be adjusted for. However, geographic and racial/ethnic stratifications were analyzed as proxies to partially capture social and structural disparities. Future studies should incorporate linked datasets to better adjust for these variables.

### Statistical analysis

To analyze national temporal trends in CKD-related mortality from 1999 to 2020, we extracted AAMRs per 100 000 population by year, sex, race/ethnicity, state, and urban–rural status with 95% CIs. AAMRs were calculated by standardizing CKD-related deaths to the year 2000 US population^[21]^. The Joinpoint Regression Program (Joinpoint V 5.0.2, National Cancer Institute) was used to determine the APC with 95% CI in mortality rates^[22]^. This method identifies significant changes in the AAMRs over time by fitting log-linear regression models where temporal variation occurred. APCs were considered increasing or decreasing if the slope describing the change in mortality was significantly different from zero using two-tailed *t*-testing. Statistical significance was set at *P* < 0.05.

### Declaration of artificial intelligence use

Artificial intelligence (AI) was used solely for language editing purposes. We utilized the free version of ChatGPT with the following prompt: “Check language accuracy and ensure academic tone.” No patient data or identifiable information was input into the tool at any stage. The AI was not involved in data analysis, interpretation, or generation of original content. This use complies with the TITAN Declaration of AI Use^[23]^.

## Results

### CKD-related mortality among older adults

In older adults (aged ≥65), a total of 1 572 075 CKD-related deaths have occurred between 1999 and 2020 (Supplemental Digital Content Table 1, available at: http://links.lww.com/MS9/A930).

### CKD-related AAMR annual trends

**Figure 1.**
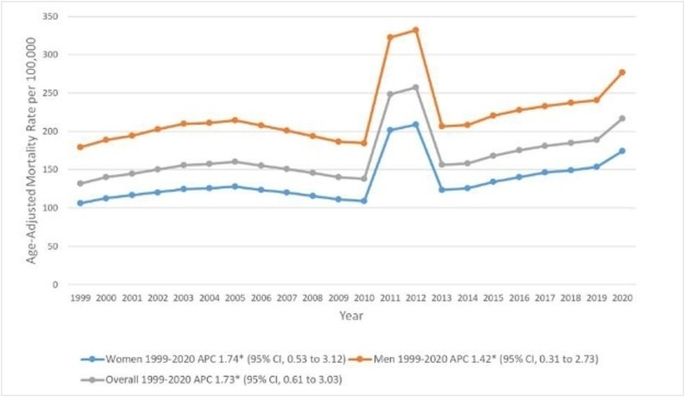

Between 1999 and 2020, the overall AAMR for older individuals rose from 132.0 to 216.6 [APC, 1.7 (95% CI, 0.6–3.0)] (Fig. 1, Supplemental Digital Content Table 2, available at: http://links.lww.com/MS9/A931 and Supplemental Digital Content Table 3, available at: http://links.lww.com/MS9/A932).

### CKD-related AAMRs classified by sex

Throughout the study timeframe, AAMR in older men remained greater than AAMR in older females. Also, during this period, there was a similar increase in CKD-related mortality in both older men [APC, 1.4 (95% CI, 0.3–2.7)] and older females [APC, 1.7 (95% CI, 0.5–3.1)] (Fig. 1, Supplemental Digital Content Table 2, available at: http://links.lww.com/MS9/A931 and Supplemental Digital Content Table 3, available at: http://links.lww.com/MS9/A932).

### CKD-related AAMRs classified by race

NH American Indian or Alaska Native older adults had the second-highest overall AAMR [188.8 (95% CI, 185.1–192.6)], after NH Black or African American older adults [312.3 (95% CI, 311.1–313.6)].

**Figure 2.**
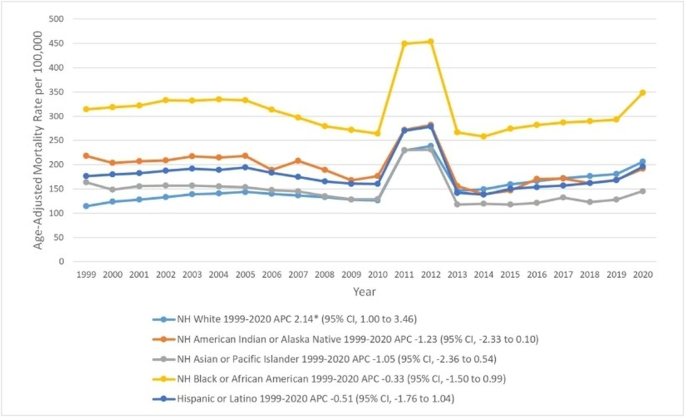

Hispanic or Latino older adults [178.5 (95% CI, 177.5–179.6)], non-Hispanic (NH) White older adults [157.3 (95% CI, 157.1–157.6)], and NH Asian or Pacific Islander older adults [143.9 (95% CI, 142.6–145.2)]. During the study interval, AAMR decreased among all races/ethnicities except NH White older adults, in whom the AAMR increased [APC, 2.1 (95% CI, 1.0–3.5)], followed by NH American Indian or Alaska Native older adults [188.8 (95% CI, 185.1–192.6)], Hispanic or Latino older adults [178.5 (95% CI, 177.5–179.6)], NH White older adults [157.3 (95% CI, 157.1–157.6)], and NH Asian or Pacific Islander older adults [143.9 (95% CI, 142.6–145.2)]. During the study interval, AAMR decreased among all races/ethnicities except NH White older adults in whom the AAMR increased [APC, 2.1 (95% CI, 1.0–3.5)] (Fig. 2, Supplemental Digital Content Table 2, available at: http://links.lww.com/MS9/A931 and Supplementary Digital Content Table 4, available at: http://links.lww.com/MS9/A933).

### CKD-related AAMRs classified by region

**Figure 3.**
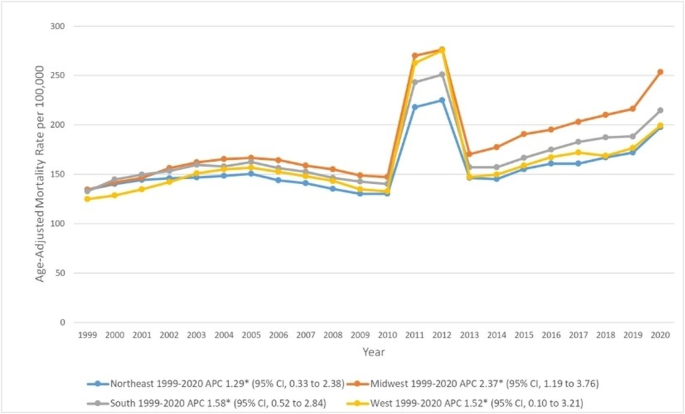

From 1999 to 2002, AAMR was higher in metropolitan areas compared to non-metropolitan areas. However, from 2007 to 2020, AAMR in non-metropolitan areas remained greater than AAMR in urban areas. Taking the study period as a whole, the AAMR increase in non-metropolitan regions [APC, 2.4 (95% CI, 1.3–3.6)] was greater than the increase in metropolitan areas [APC, 1.6 (95% CI, 0.4–2.9)] (Fig. 3, Supplemental Digital Content Table 2, available at: http://links.lww.com/MS9/A931 and Supplemental Digital Content Table 5, available at: http://links.lww.com/MS9/A934).

**Figure 4.**
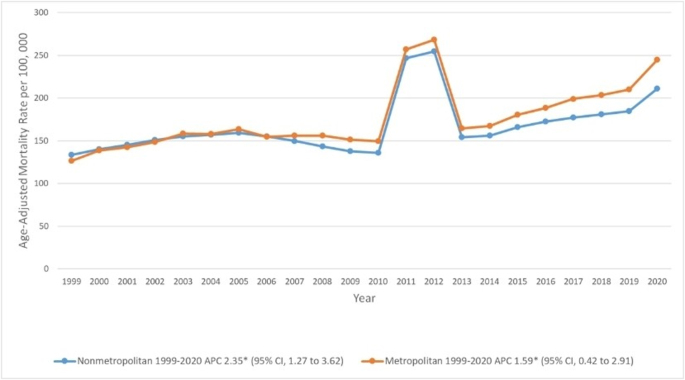

Over the study interval, CKD-related mortality increased in all regions, with the highest increase observed in the Midwestern region [APC, 2.37 [95% CI, 1.19–3.76)] (Fig. 4 and Supplemental Digital Content Table 7, available at: http://links.lww.com/MS9/A936).

AAMR varied from state to state, with the lowest AAMR reported in Arizona [112.6 (95% CI, 111.1–114.1)] and the highest AAMR reported in West Virginia [230.6 [227.0–234.3)]. CKD-related mortality was significantly higher in the top 90th percentile states: Ohio, South Carolina, North Carolina, Indiana, North Dakota, and West Virginia than in the bottom 10th percentile states: Arizona, Florida, Nevada, Utah, Montana, and Wyoming (Supplemental Digital Content Table 6, available at: http://links.lww.com/MS9/A935).

## Discussion

This study highlights a concerning rise in CKD-related mortality among older adults in the United States over the past two decades. While some national reports have documented a decline in overall CKD-related mortality^[24]^, our findings suggest a divergent trend within the ageing population. One plausible explanation is the increased prevalence and earlier onset of CKD in the general population, resulting in a larger cohort of individuals living longer with the disease^[25]^. This trend may be closely tied to the rising prevalence of key risk factors such as diabetes, hypertension, and obesity^[10–12]^. Additionally, advances in healthcare may have extended the lives of CKD patients, allowing more to reach older ages and advanced stages of the disease before death^[26,27]^. Given the natural decline in estimated glomerular filtration rate with age, the overall aging of the US population is inevitably associated with a larger burden of CKD and, consequently, higher mortality in older adults^[28–30]^. These individuals are more likely to experience cumulative comorbidities and complications, which may contribute to increased mortality^[31]^. This finding is consistent with prior studies indicating that rapid declines in kidney function and diminishing benefits of dialysis in older patients are associated with heightened cardiovascular and all-cause mortality^[32,33]^.

Our findings also reveal persistent and significant disparities in CKD-related mortality. NH Black and American Indian/Alaska Native older adults consistently experienced the highest AAMRs, whereas White and Asian/Pacific Islander individuals had the lowest. This is consistent with literature revealing high mortality among blacks^[34,35]^. These disparities mirror long-standing systemic inequities in healthcare access, including lower rates of early CKD screening, delayed diagnosis, limited referral to nephrologists, and reduced access to transplantation for minority groups^[36,37]^. Black patients, for instance, are less likely to receive kidney transplants and more likely to experience delayed diagnosis and treatment, contributing to worse outcomes^[38–40]^. Interestingly, while the APC in mortality decreased for most racial and ethnic groups, it increased markedly among NH White older adults, nearly doubling between 1999 and 2020. This alarming trend warrants further investigation. It is possible that some of these deaths reflect broader sociocultural issues – such as the rise in “deaths of despair” related to substance use, economic stress, and untreated mental health conditions among older White individuals^[41]^. The increasing prevalence of obesity and other lifestyle-related risk factors may also be contributing to this pattern^[42]^.

Geographic disparities were also evident. Older adults residing in non-metropolitan areas experienced consistently higher CKD mortality rates compared to their urban counterparts. This is likely driven by limited access to nephrology services, fewer healthcare resources, and socioeconomic disadvantages in rural settings^[43–45]^. CKD management requires extensive resources, including routine monitoring, dietary and medication support, frequent follow-up visits, dialysis, and in some cases, transplantation^[46]^. When access to these services is restricted, the likelihood of delayed intervention and higher mortality increases substantially. This places an additional economic and structural burden on already underserved communities and underscores the importance of equitable healthcare delivery^[47–49]^.

Gender-based differences were also observed, with older men experiencing higher mortality than women. This is consistent with existing literature, which suggests that men are more likely to progress to end-stage renal disease and are less likely to engage in preventive or routine healthcare practices^[50,51]^.

These findings emphasize the need for augmenting efforts to prevent and treat CKD in older adults and the underserved population. Cardiovascular disease remains a leading cause of death among CKD patients, yet management is often complicated due to the exclusion of CKD patients from many clinical trials^[52–54]^. As a result, providers may underuse evidence-based therapies out of concern for worsening renal function^[55]^. Nevertheless, accumulating evidence supports the cautious use of proven therapies such as sodium-glucose cotransporter-2 inhibitors and non-steroidal mineralocorticoid receptor antagonists, which offer cardiovascular and renal protection^[56]^.

To address the rising burden of CKD-related mortality among older adults, particularly within underserved populations, policy interventions must extend beyond general prevention efforts. One critical step involves expanding Medicare coverage to include routine CKD screening for high-risk individuals, such as those with diabetes or hypertension, to facilitate earlier detection and timely intervention. Notably, over 70% of Medicare beneficiaries with laboratory indicators of CKD lacked a corresponding diagnostic code in their medical records, underscoring a significant gap in documentation and disease recognition^[57]^. Improving access to nephrology care also requires addressing the ongoing shortage of nephrologists, particularly in non-metropolitan areas where disparities are most evident^[58,59]^. Policies aimed at expanding nephrology capacity should focus on standardizing training programs, increasing access to dialysis and kidney care services, and optimizing the current workforce through the adoption of multidisciplinary nephrology care teams^[60]^. Furthermore, implementing standardized care pathways and clinical decision-support tools – especially those informed by machine learning – can promote the delivery of evidence-based care across diverse clinical environments. Integrating social risk factor screening into electronic health records may also support more holistic, equity-oriented care planning. AI-driven models offer substantial promises for enhancing early CKD detection by identifying high-risk individuals, predicting disease trajectories, and informing more efficient allocation of healthcare resources – particularly in underserved, rural areas where access and outcomes remain suboptimal^[61,62]^. Health systems should be incentivized to invest in preventive services and coordinated care models, which have the potential to improve outcomes and reduce disparities across populations.

Although this study did not incorporate AI-based methods, future research could explore the development and validation of predictive algorithms or clinical decision-support tools specifically tailored to the aging population, with the goal of advancing early diagnosis and optimizing management of CKD in high-risk groups.

### Limitations

This study has several limitations. Mortality data were based on death certificates, which may underreport or misclassify CKD, especially when not listed as the underlying cause of death. Physicians may differ in how thoroughly they report comorbid conditions, leading to inconsistencies in CKD attribution and while the use of multiple cause of death coding allowed for a more inclusive capture of CKD-related mortality, it also introduces variability in the degree to which CKD contributed to death in each case, limiting causal interpretation. Key confounders such as socioeconomic status, comorbidities, and healthcare access were unavailable in the dataset, limiting the ability to adjust for these factors.

## Conclusions

In summary, this study examined trends in CKD-related mortality among US adults aged 65 and older from 1999 to 2020. Our findings show a sustained increase in AAMRs over the study period, with disproportionately higher rates observed among NH Black adults – more than double those of their White counterparts – and substantial variation across US regions. These disparities suggest persistent structural and geographic inequities in CKD diagnosis, management, and care access.

Future research should focus on identifying the drivers of regional disparities and developing predictive models that incorporate social determinants of health. In parallel, public health policies should prioritize targeted screening programs and culturally informed prevention strategies in high-risk communities, especially among racially minoritized and socioeconomically disadvantaged older populations.

## Data Availability

The data used in this research are publicly available at CDC WONDER database.
